# Re: “Amniotic fluid from healthy term pregnancies does not harbor a detectable microbial community” (2018) 6:87, https://doi.org/10.1186/s40168-018-0475-7

**DOI:** 10.1186/s40168-019-0642-5

**Published:** 2019-02-12

**Authors:** M. S. Payne, J. A. Keelan, L. F. Stinson

**Affiliations:** 1 Division of Obstetrics and Gynaecology, School of Medicine, University of Western Australia, 35 Stirling Highway, Crawley, Western Australia Australia; 2 School of Biomedical Sciences, University of Western Australia, 35 Stirling Highway, Crawley, Western Australia Australia

**Keywords:** Amniotic fluid, Pregnancy, Bacteria, Viruses, Microbiome

## Abstract

A recent publication by Lim et al. 2018 (Amniotic fluid from healthy term pregnancies does not harbor a detectable microbial community) strongly concluded that the microbiome of amniotic fluid primarily originates from reagent contamination. However, upon closer inspection of the methods used and data presented in this study, in particular the supplementary data, such conclusions do not appear to be supported by the results. We outline such methodological/data interpretation concerns and invite the authors to discuss these.

## Main text

Dear Professor Ravel,

We recently read with great interest an article published by Lim, Rodriguez and Holtz in Microbiome entitled “Amniotic fluid from healthy term pregnancies does not harbor a detectable microbial community”. However, as scientists working in the same field (molecular microbiology and obstetrics), we were very surprised at some of the conclusions drawn in light of the methods and results that were presented in the paper.

The article appears to rather definitively conclude that “amniotic fluid of healthy term pregnancies has negligible bacterial biomass” and that “the term infant is not normally exposed to bacterial or viral populations in the immediate pre-birth interval”. These are very strong conclusions, especially considering this contradicts both of the previously published studies on the amniotic fluid bacterial microbiome, one of which attempted to control for contamination [1, 2]. We would be greatly appreciative if you could please invite the authors to comment on the following concerns that we have:

## Methods

1) Sample collection: “Amniotic fluids were obtained in a sterile fashion at the time of C-section by aspirating through intact amniotic membranes. The amniotic fluid was then spun at 1620g for 5 min at 4 °C. Fluid was then placed into conical tubes and stored at − 80 °C.”

Why were the samples initially spun at 1620×*g* for 5 min and the supernatant then frozen and used for the microbial profiling? Although this is a slow spin, it will certainly still pellet some bacterial cells present, in addition to human cells that may either have bacterial cells adhered to the cell surface or contain obligate intracellular bacteria, such as Chlamydia spp. for example. The fact that the supernatant from this spin step was then used as the sample that was analysed in the study potentially completely confounds the data as samples that were apparently negative for bacterial DNA may have had any cells present removed during this initial spin. In addition, none of the control samples were also exposed to this pre-analysis centrifugation step.

2) Bacterial 16S rRNA gene qPCR

Taqman Fast Advanced PCR mastermix is not a SYBR green mastermix; it is designed for use with hydrolysis probes. More importantly though, as 16S rRNA gene copy number varies between and within bacterial species, using a standard curve generated from a single organism such as *E. coli* will give loosely semi-quantitative data at best. It also appears that no human DNA-only controls were used in this assay. These controls are necessary as in low microbial biomass clinical samples, the amount of human DNA present often drastically outweighs that of microbial DNA, and many 16S rDNA primers will bind to human DNA with varying efficiency, making it almost impossible to draw meaningful results from low titre samples without use of appropriate controls [3].

3) “Seven samples were omitted from further analyses as they contained less than 5000 16S rRNA gene sequencing reads (five amniotic fluids, one water, and one buffer)”

Why were these samples omitted? Five thousand reads are still plenty in a low microbial biomass sample.

## Results and discussion

The authors have only included OTUs in their analyses that originated from bacterial reads which were detected in the amniotic fluid samples and not in the blank extraction or PCR controls, regardless of the levels at which they were detected (Figure 2). The OTU table presented in additional file 3 suggests that this is not the correct approach to take. Numerous OTUs have been identified in very low levels in the blank controls, but are also present in very high levels in the amniotic fluid samples (e.g. OTUs 2, 4, 5, 8, and 17). For example, OTU 4 only accounted for 0–10 reads in the controls, but gave as many as 88,185 reads in the amniotic fluid samples. If the presence of these OTUs in amniotic fluid samples was merely due to contamination, we would expect to see similar levels of these taxa in both the samples and controls. These details were not discussed in the manuscript and were instead present only within the supplementary data. In cases like the example we provide, why were the low/negligible number of reads present in the controls not subtracted from those present in the samples and these data presented in the analyses (in particular, reads seen in the controls should be subtracted from samples associated with that same DNA extraction)?

In addition, when you examine the OTUs assigned to the buffer and water controls, there are very distinct differences in the number of reads between them. For example, ‘water 7’ has 4448 reads assigned to OTU 11, while none of the other water controls have this. Similarly, ‘buffer 7’ and ‘buffer 8’ have 520 and 1719 reads respectively assigned to OTU 3, yet ‘buffer 9’ has 0. Apparent contamination from kit reagents and PCR mastermixes should be reasonably consistent across negative controls, as the contaminants are derived from a central source. The variation between controls seen in this study could more likely be explained through user-introduced contamination and, if this is the case, confounds the results even further as there may be random incidents of contamination introduced across the samples that are undetectable using controls. The use of a spin column kit could support this theory in that individual tubes/lids, etc., are constantly opened and closed by non-sterile gloves and in many cases are centrifuged during the extraction process un-capped (we can only speculate on how the authors carried out the extractions though, as no details are provided beyond the kit used, nor how many extraction runs were performed or if only one run was performed with all controls on the same run).

The authors have also concluded quite strongly that the amniotic fluid microbiome is indistinguishable from contamination controls. However, their data by no means suggests this. Examination of the PCoA plots and supplementary data do not support this conclusion. For instance, some amniotic fluid samples cluster more closely with stool than with buffer controls. Furthermore, as discussed above, the supplementary material in additional file 3 shows that the amniotic fluid samples were quite distinct from the controls and the authors even present the data showing numerous OTUs associated with bacterial reads that were present in amniotic fluid samples only, suggesting that at the very least, low levels of bacterial DNA are present in the amniotic fluid of uncomplicated term pregnancies prior to delivery. Whether this DNA represents viable cells and is clinically relevant is not known as additional work is required to ascertain this.

Finally, the two paragraph discussion makes little attempt to provide an explanation for the results, particularly so in light of the very interesting data they presented on viruses showing that all but one viral read from the amniotic fluid were associated with bacteriophage RNA/DNA. This adds further weight to the argument that there could be a viable bacterial microbiome in the amniotic fluid, as phage can only replicate within bacterial cells and may be present as a result of the release of phage progeny from a lysed bacterial cell.

Yours Sincerely,

Dr. Matthew Payne (PhD, FASM)

Professor Jeffrey Keelan (PhD, FSRB)

Ms. Lisa Stinson (MSc, 3rd year PhD candidate)
